# Music influences vividness and content of imagined journeys in a directed visual imagery task

**DOI:** 10.1038/s41598-021-95260-8

**Published:** 2021-08-06

**Authors:** Steffen A. Herff, Gabriele Cecchetti, Liila Taruffi, Ken Déguernel

**Affiliations:** 1 École Polytechnique Fédérale de Lausanne, INN 115, 1015 Lausanne, Switzerland; 2 The MARCS Institute for Brain, Behaviour and Development, Western Sydney University, Sydney, Australia; 3 Music Department, Durham University, Durham, UK; 4 CNRS, Centrale Lille, UMR 9189 CRIStAL, Université de Lille, F-59000 Lille, France

**Keywords:** Psychology, Human behaviour

## Abstract

Directed, intentional imagination is pivotal for self-regulation in the form of escapism and therapies for a wide variety of mental health conditions, such anxiety and stress disorders, as well as phobias. Clinical application in particular benefits from increasing our understanding of imagination, as well as non-invasive means of influencing it. To investigate imagination, this study draws from the prior observation that music can influence the imagined content during non-directed mind-wandering, as well as the finding that relative orientation within time and space is retained in imagination. One hundred participants performed a directed imagination task that required watching a video of a figure travelling towards a barely visible landmark, and then closing their eyes and imagining a continuation of the journey. During each imagined journey, participants either listened to music or silence. After the imagined journeys, participants reported vividness, the imagined time passed and distance travelled, as well as the imagined content. Bayesian mixed effects models reveal strong evidence that vividness, sentiment, as well imagined time passed and distances travelled, are influenced by the music, and show that aspects of these effects can be modelled through features such as tempo. The results highlight music’s potential to support therapies such as Exposure Therapy and Imagery Rescripting, which deploy directed imagination as a clinical tool.

## Introduction

Imagining objects, stories, or scenarios plays an important role in action planning^1^, simulating past and future events^2^, self-regulation in the form of escapism^3^, and the therapy of anxiety and stress disorders, as well as phobias^4–6^. In particular, in-sensu techniques, in which patients imagine traumatic or anxiety-inducing scenarios, are an essential part of Cognitive Behaviour Therapy (CBT)—including Exposure Therapies—and have proven very effective for example in the treatment of Post-Traumatic Stress Disorder^7^. Current evidence-based therapies such as Imagery Rescripting^8^ require patients to imagine a traumatic event and then imagine an intervention that changes the outcome, providing relief and a sense of empowerment^9^. Thus, enabling subtle control over imagined content could provide great therapeutic value, both for imagination as a form of self-regulation as well as its use in clinical settings. Specifically, multimodal mental imagery, where information in one domain (e.g., the auditory) induces imagination in another (e.g., the visual), is a commonly observed phenomenon, and its potential to support therapy has been highlighted before^10^. However, due to the methodological difficulties in reliably inducing, manipulating, and measuring imagination in empirical studies, our understanding of this intricate process is still limited.

Music in particular has been proposed as a promising tool to explore imagination, as more than 70% of music listeners indicate that they experience visual imagery when listening to music^11–13^. This is consistent with functional neuroimaging research showing activation of the visual cortex during music listening with eyes closed^14–16^. Indeed, visual imagery has recently been identified as one of the main sensory modalities of music-evoked mental experiences such as mind-wandering^17,18^. This suggests that music functions as a reliable inducer of imagination and could be a promising tool for imagination-based therapies. Content-wise, the words listeners use to describe the experience of music-induced imaginations are similar to those observed in other media (e.g., reading), and are characterised as a form of ‘escaping reality’^3,19–21^. Indeed, the importance of ‘escapism’ as a cognitive strategy for self-regulation has been highlighted by the recent global COVID-19 pandemic, which saw a surge in video, music, book, and video game consumption^22^. However, a systematic approach to empirically measure music-induced imagination remains elusive as “at present, […]a systematic phenomenology of music-evoked visual imagery has yet to be provided”^23^(also see^24^).

Seminal work by Koelsch et al.^25^ recently provided empirical evidence that listening to music affects the thought process in mind-wandering. Specifically, 62 participants listened to three sad and three heroic excerpts, and, after each excerpt, were asked a series of questions about their thoughts. Though not explicitly directed to do so, in most cases participants began to mind-wander and imagine content other than the music they were listening to. Results further showed that music influenced the imagined content of listeners, as participants reported more positive non-directed thoughts after listening to heroic compared to sad music. The link between music and imagined content has received additional support from an earlier study also showing that music can affect the frequency, content, and neural correlates of mind-wandering episodes whilst the music’s tempo plays an important role in modulating such mental experiences^18^. However, these studies focus on spontaneously evoked mind-wandering, sometimes even specifically defined as task-unrelated thoughts^26^, rather than goal-directed, intentional imagination. This is important to note, because current cognitive therapies specifically rely on the use of goal-directed, intentional imagination in which actively shaping the content of an imagined scenario to achieve an end goal itself is the task^10^ (for a review see^5^)*.* Examples for such goal-directed creative imagination tasks include habituating to an imagined adverse stimulus during exposure therapy^27^, changing the sequence of events during imagery rescripting therapy^8^, or mentally simulating a situation in order to prepare^28,29^. How to theoretically conceptualise the relationship between spontaneous mind-wandering and directed imagination is currently unclear. Directed imagination can be thought of as a form of spontaneous mind-wandering under additional constraints^30^, or as a more modular, distinct aspect of it^31^. Much empirical evidence points towards an understanding of imagination as a form of ‘weak-perception’^5^, which would likely favour a continuous, rather than categorical distinction between spontaneous and directed imagination. Important for the present study is that, either way, present theoretical frameworks of mind-wandering do not allow to straightforwardly generalise findings from cases where imagination was spontaneous and non-task-related to scenarios in which directed imagination itself is the task. Thus, although prior research has established a general link between music and spontaneously evoked mind-wandering, whether this link holds in the specific case of intentional, directed imagination as for example utilised in behavioural therapy requires further scientific attention.

In the context of therapies as well as other directed imagination tasks, such as role playing^32^, it is of particular relevance to investigate means to influence the vividness as well as the sentiment of the directed imagined content. This is because imagery vividness can dynamically change within individuals^33^, yet increased vividness of imagined content shows a beneficial effect on exposure treatment outcome^34^, and imagery rescripting relies on specifically manipulating the sentiment of imagined scenes and narratives^8^. As mentioned above, prior research has shown that music interacts with the imagined content during spontaneously evoked mind-wandering^35^. However, whether music can be used to systematically influence vividness and sentiment in a directed imagination task is subject of the present study. As the imagined content in a directed imagination task is strongly contextualised (e.g., ‘imagine crossing a road with two lanes’), investigating directed imagination also allows exploring the effect of music on specific and task-related measures beyond vividness and sentiment, such as simulated physical properties of the imagined content.

Prior characterisations of undirected mind-wandering whilst listening to music often involves a dissociation from the body^21^, with imagined landscapes, autobiographical scenes as well as film-like narratives^13,25^, which suggests that music affects imagined (or simulated) position in space and time. This is also important to consider when investigating directed imagery—rather than non-directed mind-wandering—as prior studies have shown that listeners can associate music with physical space and imagined bodily motions^36^. Importantly, there is evidence that the perception of space and time during imagination exhibits similar psychological properties to the perception of physical space and physical time^37,38^. For instance, professional musicians^1^ and athletes^39–41^ use intentional, directed imagery to practise movements to enhance skill acquisition and performance accuracy. Fundamental principles of human motion such as Fitts law, which describes the trajectory of human movements as a function of the width of an object and distance to it, also still apply to imagined movements^37,42^. Similarly, eye movements during imagined visual sceneries are similar to those observed when scanning real visual sceneries^43^. These studies demonstrate that the sense of position in time and space remains largely active during imagination, which in turn provides a gateway to empirically assess imagination induced by music. Specifically, this means that deploying a directed imagination task that requires participants to imagine scenarios that involve the passing of time and displacement in space would allow for comparably concrete measurements of imagination—despite the elusive nature of imagination at large—by assessing imagined passing of time and imagined distance.

Here, we investigate whether music can induce and manipulate directed imagination, and measure observable features of this effect in terms of vividness and sentiment of the imagined content, and imagined orientation in time and space, whilst controlling for potential effects of tempo^18^ and musical expertise^44^. If music would show systematic effects on imagination here, then this would demonstrate that music has a direct influence on the creative thought processes underlying directed imagination. In turn, this would provide empirical support for the usage of music in therapies that rely on directed imagination, such as exposure therapy and imagery rescripting, and would motivate future research into establishing a link between specific musical and acoustic features, and their respective effects on imagination.

**Figure 1:**
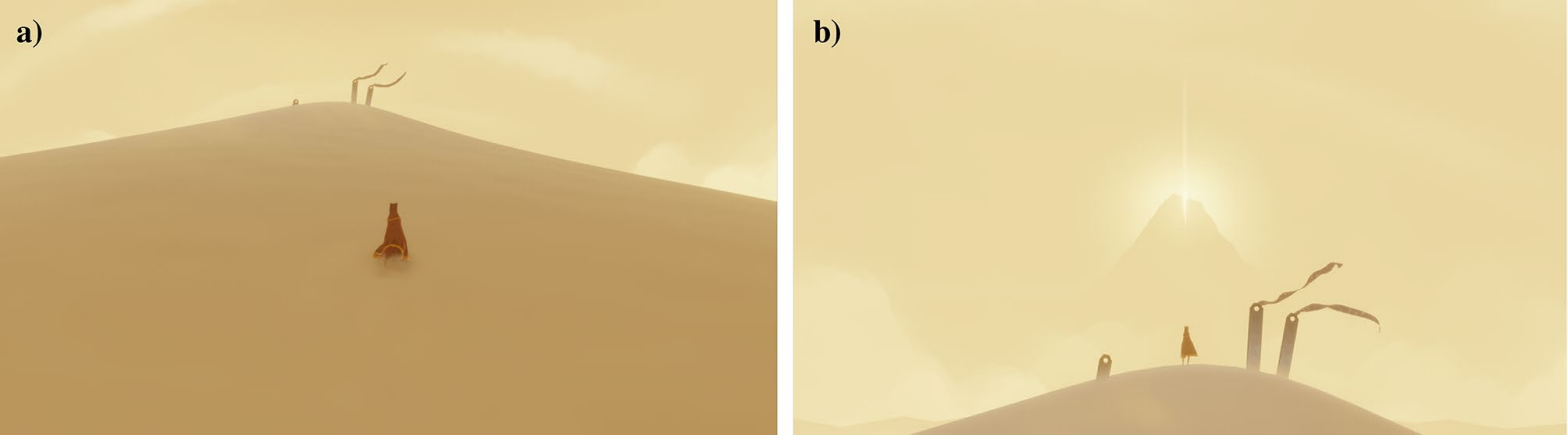
Images from the visual inducer video. A figure ascending a small hill was visible (**a**). Shortly after the figure reached the top of the small hill, a large mountain (**b**), barely visible, appeared in the far distance, and participants were instructed to close their eyes and imagine a continuation of the journey towards the landmark.

In the present study, 100 participants engaged in a task that required closing their eyes and imagining a continuation of a journey started in a visual inducer video (Fig. 1). In each trial, participants performed the task either in silence, or whilst hearing one of six auditory inducers in the form of music. The musical stimuli comprise 3 different musical pieces (J. S. Bach, Chorale ‘O Haupt voll Blut und Wunden’; C. Debussy, ‘Tarantelle Styrienne’; R. Rodgers, ‘My Favorite Things’), with two distinct renditions of each (see Supplement S1). At the end of each trial, participants answered questions about the vividness of their imagination, the amount of imagined time passed as well as imagined distance travelled, and also provided a detailed description of their imagination.

## Aims and hypotheses

To deepen our understanding of directed imagination, a process used in cognitive therapies, as well as self-regulation, this study has two main aims: (a) To establish a novel paradigm to explore directed imagination through vividness, sentiment, and simulated orientation in time and space, in order to better understand the process of imagination and explore means to influence imagined content through external inducers; and (b) to specifically investigate whether music can be used to influence directed imagination. To this end, it is hypothesised that:

1. The musical auditory inducers induce more vivid imaginations than silence (supported)
2. The sentiment of the imagined content differs based on the auditory inducer condition (supported)
3. Differences between induced imagined time and distance can be observed between auditory inducers (supported)
4. The effect of the auditory inducers on imagination is mediated by tempo (supported)
5. Musical expertise is reflected in the measures of imagined content (not supported)

## Results

The data was analysed using Bayesian mixed effects models which allow to model individual responses whilst controlling for dependencies within the data as well as cross-random effects, for example, between participants and trial numbers^45^. The models were implemented in R^46^ using the brms package^47,48^. All continuous variables were scaled to have a mean of 0 and a standard deviation of 1, and all models were provided with a weakly informative prior (a t-distribution with a mean of 0, a standard deviation of 1, and 3 degrees of freedom)^49^, and ran with 1.000 warm-ups and 10.000 iterations on four chains. For each hypothesis we report the model coefficients (*β*) relevant to the specific hypotheses, the estimated error of this coefficients (*EE*_*β*_), as well as the evidence ratio in favour of a given hypothesis (*Odds*_*β*_). For conveniences we denote effects than can be considered ‘significant’ under an alpha level of 5% with * (i.e., evidence ratio ≥ 19; see^50^) All data and scripts used for analysis are available in Supplement S3 or through https://osf.io/w2d8k/.

### The musical auditory inducers induce more vivid imaginations than silence

**Figure 2:**
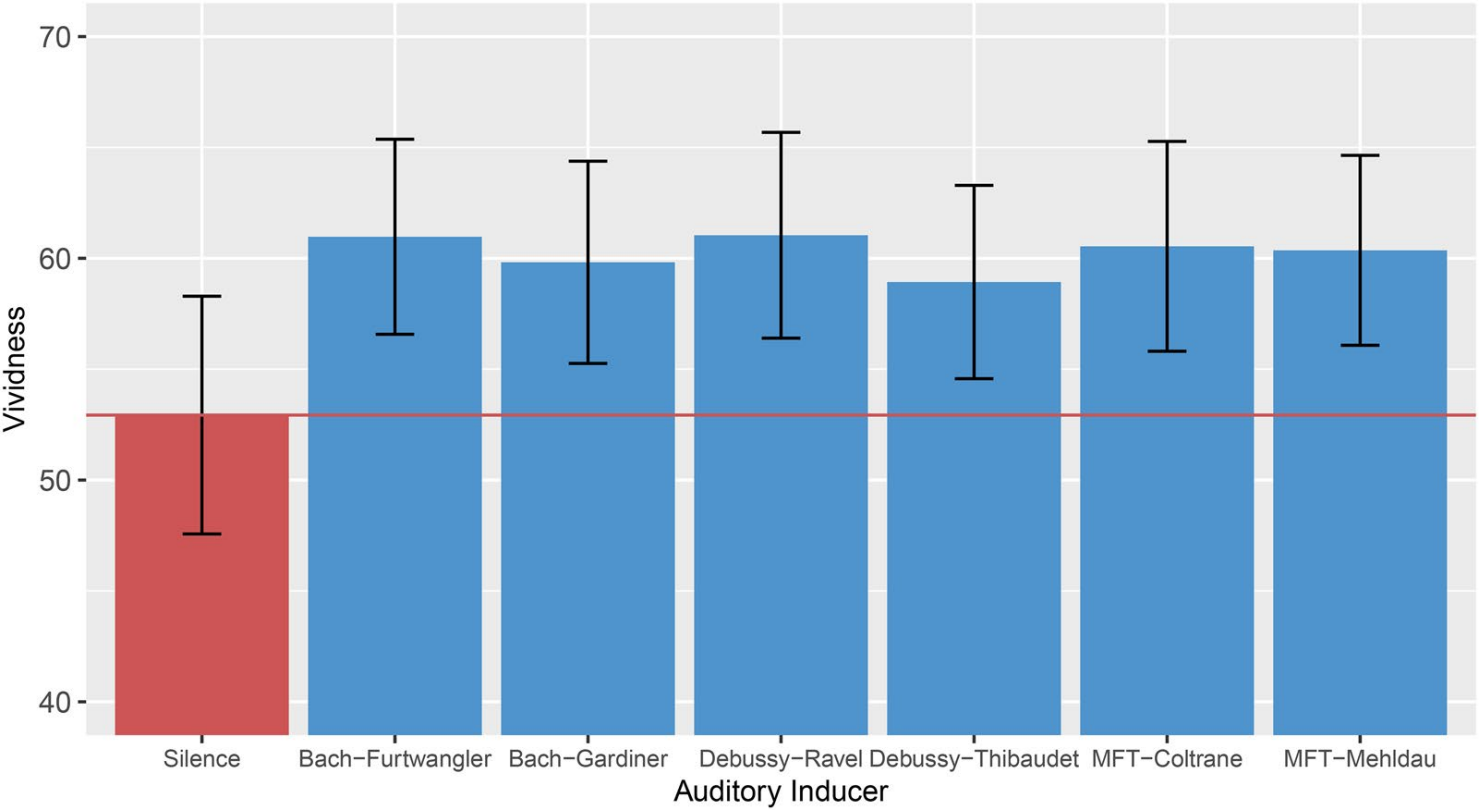
Average vividness by auditory inducer condition. All three musical auditory inducers, with two different renditions each, led to higher vividness than the silent control condition. The red line shows the mean of the silent control condition. Error bars show 95% CIs.

Participants made full use of the spectrum of the 100-point vividness scale (*M* = 59.23, *SD* = 23.67, *Min* = 1, *Max* = 100). A Bayesian mixed effects model was used to predict Vividness using Auditory Inducer (factor labelled with composer followed by performer, conductor, or orchestrator, with levels: Bach-Furtwängler, Bach-Gardiner, Debussy-Ravel, Debussy-Thibaudet, MFT-Coltrane, MFT-Mehldau, Silence*,* see “Method” section) as predictor, with a random intercept for participant and trial number. As seen in Fig. 2, all auditory inducers led to higher vividness ratings than the silence condition. Specifically, strong evidence was observed for Bach-Furtwängler (*β* = 0.34, *EE*_*β*_ = 0.1, *Odds* (*β* > 0) = 4499*)*,* Bach-Gardiner (*β* = 0.29, *EE*_*β*_ = 0.1, *Odds* (*β* > 0) = 579.65*)*,* Debussy-Ravel (*β* = 0.34, *EE*_*β*_ = 0.1, *Odds* (*β* > 0) = 2768.23*)*,* Debussy-Thibaudet (*β* = 0.26, *EE*_*β*_ = 0.1, *Odds* (*β* > 0) = 193.59*)*,* MFT-Coltrane (*β* = 0.32, *EE*_*β*_ = 0.1, *Odds* (*β* > 0) = 1383.62*), and MFT-Mehldau (*β* = 0.31, *EE*_*β*_ = 0.1, *Odds* (*β* > 0) = 1027.57*) to induce higher vividness ratings than the silence condition. This supports the hypothesis that the musical auditory inducers induce more vivid imaginations that the silence condition. Indeed, on average, vividness ratings obtained from the music conditions were 2.7 times higher compared to vividness ratings obtained from the same respective participant during the silence condition.

### The sentiment of the imagined content differs based on the auditory inducer condition

**Figure 3:**
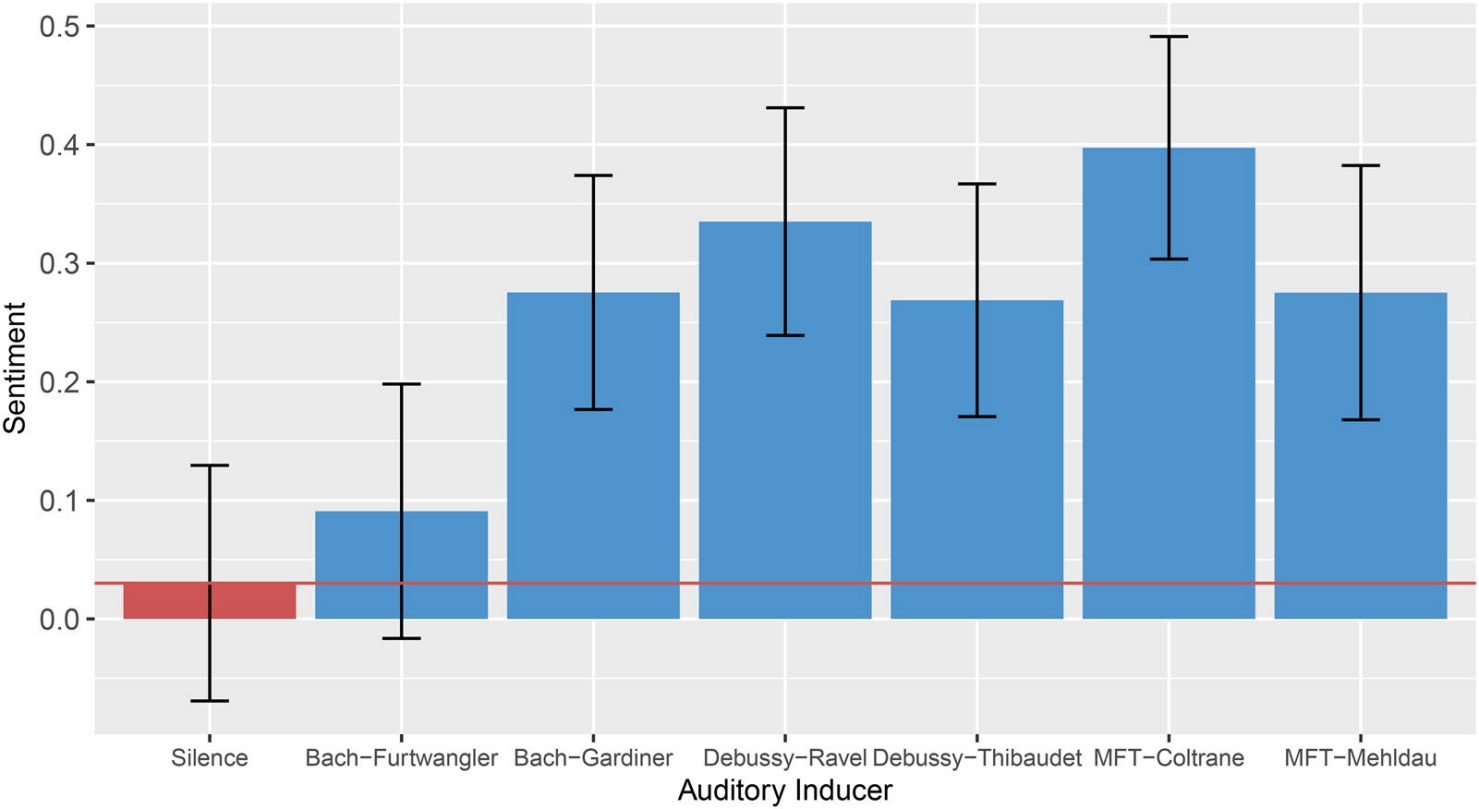
Average sentiment by auditory inducer condition. All but one music condition show significantly more positive sentiment in the description of the imagined journeys compared to the silence condition. Differences between renditions of the same musical piece were also observed. The red line shows the mean of the silent control condition. Error bars show 95% CIs.

We used NLTK^51^ and VADER^52^ to analyse the sentiment of the detailed descriptions of the imaginations, which provides an average sentiment score (the larger the value, the more positive the sentiment) for each description provided by the participants. Bayesian mixed effects models were then used to predict Sentiment*,* using the Auditory Inducer (factor with levels Bach-Furtwängler, Bach-Gardiner, Debussy-Ravel, Debussy-Thibaudet, MFT-Coltrane, MFT-Mehldau, Silence) as predictor, whilst controlling for random effects of participant and trial number. Bach-Gardiner (*β* = 0.47, *EE*_*β*_ = 0.14, *Odds* (*β* > 0) = 2768.23*)*,* Debussy-Ravel (*β* = 0.59, *EE*_*β*_ = 0.14, *Odds* (*β* > 0) > 9999*)*,* Debussy-Thibaudet (*β* = 0.46, *EE*_*β*_ = 14, *Odds* (*β* > 0) = 2768.23*)*,* MFT-Coltrane (*β* = 0.70, *EE*_*β*_ = 0.14, *Odds* (*β* > 0) > 9999*)*,* and MFT-Mehldau (*β* = 0.47, *EE*_*β*_ = 0.14, *Odds* (*β* > 0) = 2570.43*) all induce more positive sentiment in the detailed descriptions of the imagined journeys compared to the silence condition. Only Bach-Furtwängler (*β* = 0.12, *EE*_*β*_ = 0.14, *Odds* (*β* > 0) = 4.19) does not induce more positive sentiment compared to the silence condition. This can be seen in Fig. 3 by the large distance between all but one of the blue bars and the red line that indicates the silent baseline condition. Furthermore, the two My Favorite Things renditions induced different degrees of sentiment, with the MFT-Coltrane rendition inducing more positive sentiments in the descriptions of the imagined journeys compared to the MFT-Mehldau rendition (*β* = 0.23, *EE*_*β*_ = 0.14, *Odds* (*β* > 0) = 20.05*). The same was observed with the two renditions of the Bach Chorale, whereby Bach-Gardiner induced more positive sentiment (*β* = 0.35, *EE*_*β*_ = 0.14, *Odds* (*β* > 0) = 217.18*). This can also be seen in Fig. 3, by the comparably large difference between the two left-most blue columns, as well as between the two right-most two columns.

### Differences between induced imagined time and distance can be observed between the auditory inducer conditions

Due to the large spread of responses in both the imagined distance (from 1 m to 7000 km) as well as the imagined time (from 1 s to over a year) measures, all values were natural log scaled, and then participant-wise standardised by scaling to a mean of 0 and a standard deviation of 1.

Analogous to prior analyses, Bayesian mixed effects models were then used to predict Standardised Log Imagined Distance and Standardised Log Imagined Time using the Auditory Inducer (factor with levels Bach-Furtwängler, Bach-Gardiner, Debussy-Ravel, Debussy-Thibaudet, MFT-Coltrane, MFT-Mehldau, Silence) as predictor, whilst controlling for random effects of participant and trial number. Note that a preliminary analysis was conducted by modelling the data with a more complex nested model, that embeds different renditions as levels of a song condition, and different songs as levels of a binary music vs silence factor. The results were identical to those presented here. The full nested alternative models and their results can be found in the Supplement S3.

**Figure 4:**
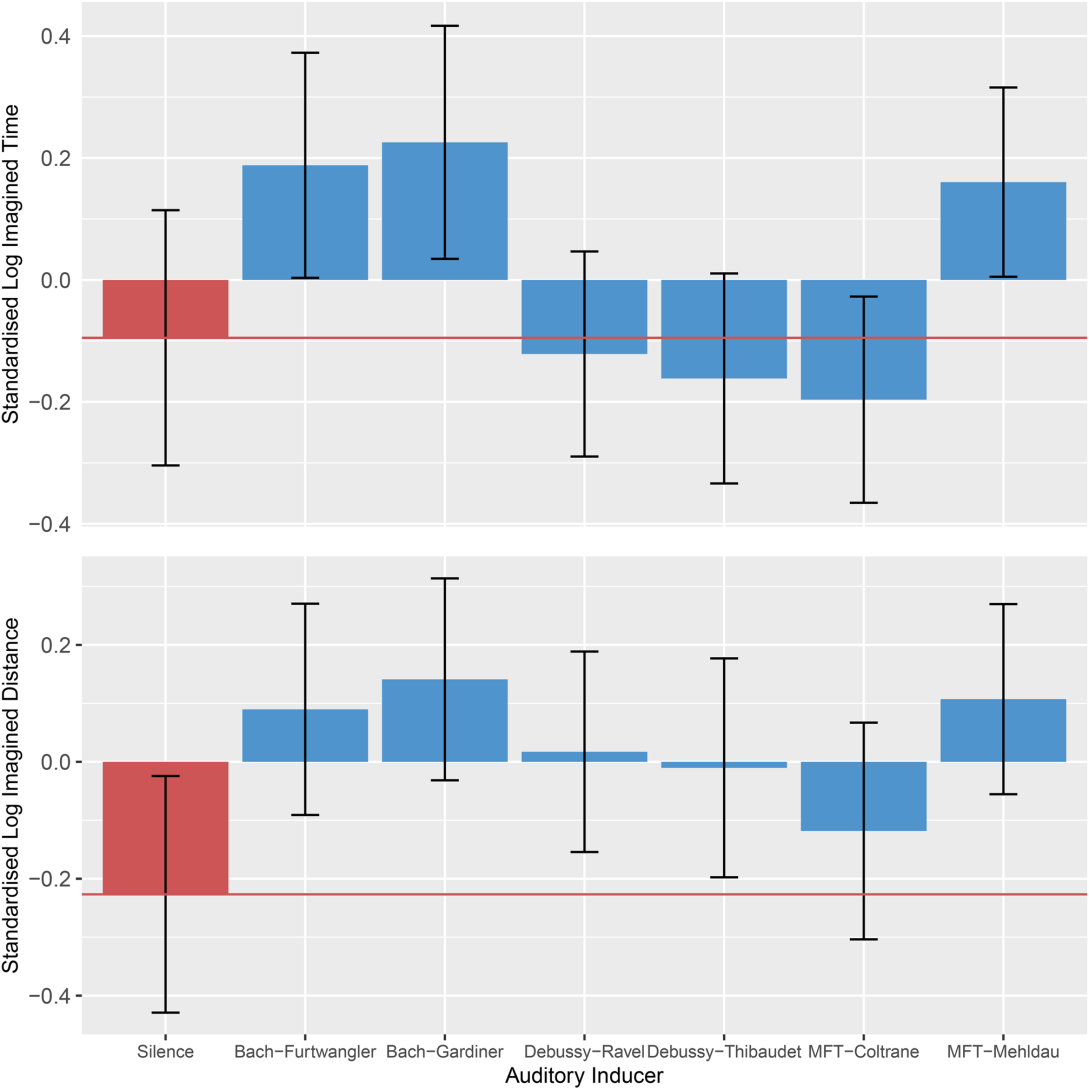
Standardised Log Imagined Time (upper panel) and Distance (lower panel). Differences between the music auditory inducers (blue) and the silence condition (red) are observed, as well as between renditions of the same inducer. For example, renditions of My Favorite Things by Coltrane and Mehldau induce different degrees of imagined distance as well as time. The red line indicates the mean of the silent control condition. Error bars show 95% CIs.

Strong evidence was obtained showing that Bach-Furtwängler (*β* = 0.31, *EE*_*β*_ = 0.13, *Odds* (*β* > 0) = 117.03*)*,* Bach-Gardiner (*β* = 0.37, *EE*_*β*_ = 0.13, *Odds* (*β* > 0) = 394.60*)*,* Debussy-Ravel (*β* = 0.25, *EE*_*β*_ = 0.13, *Odds* (*β* > 0) = 32*.*15*)*,* Debussy-Thibaudet (*β* = 0.22, *EE*_*β*_ = 0.13, *Odds* (*β* > 0) = 19.51*)*,* and MFT-Mehldau (*β* = 0.33, *EE*_*β*_ = 0.13, *Odds* (*β* > 0) = 187.48*) induced more imagined distance than the silence condition. The only condition that did not show evidence for more induced imagined distance than the silence condition was MFT-Coltrane (*β* = 0.11, *EE*_*β*_ = 0.13, *Odds* (*β* > 0) = 3.85). Furthermore, strong evidence was obtained showing that the two renditions of My Favorite Things induced different amounts of imagined distance (*β* = 0.23, *EE*_*β*_ = 0.13, *Odds* (*β* > 0) = 22.32*). This can be seen in Fig. 4, lower panel, by the large distances between the blue bars (music auditory inducers) to the red line (silence condition), as well as the large distance between the two right-most blue bars.

Strong evidence was also obtained showing that Bach-Furtwängler (*β* = 0.28, *EE*_*β*_ = 0.13, *Odds* (*β* > 0) = 57.92*)*,* Bach-Gardiner (*β* = 0.32, *EE*_*β*_ = 0.13, *Odds* (*β* > 0) = 151.54*)*,* and MFT-Mehldau (*β* = 0.25, *EE*_*β*_ = 0.13, *Odds* (*β* > 0) = 37.10*) induced more imagined time than the silence condition. Such strong evidence was not observed for the other auditory inducer conditions (all *Odds* < 3.76). However, strong evidence was observed supporting differences in induced imagined time between the two renditions of My Favorite Things (*β* = 0.36, *EE*_*β*_ = 0.13, *Odds* (*β* > 0) = 299*)*.* This can be seen in Fig. 4, upper panel, by the large distances between the two left-most as well as the far-right blue bars and the red line, and by the large difference between the two right-most blue bars.

### The effect of the auditory inducers on imagination is mediated by tempo

**Figure 5:**
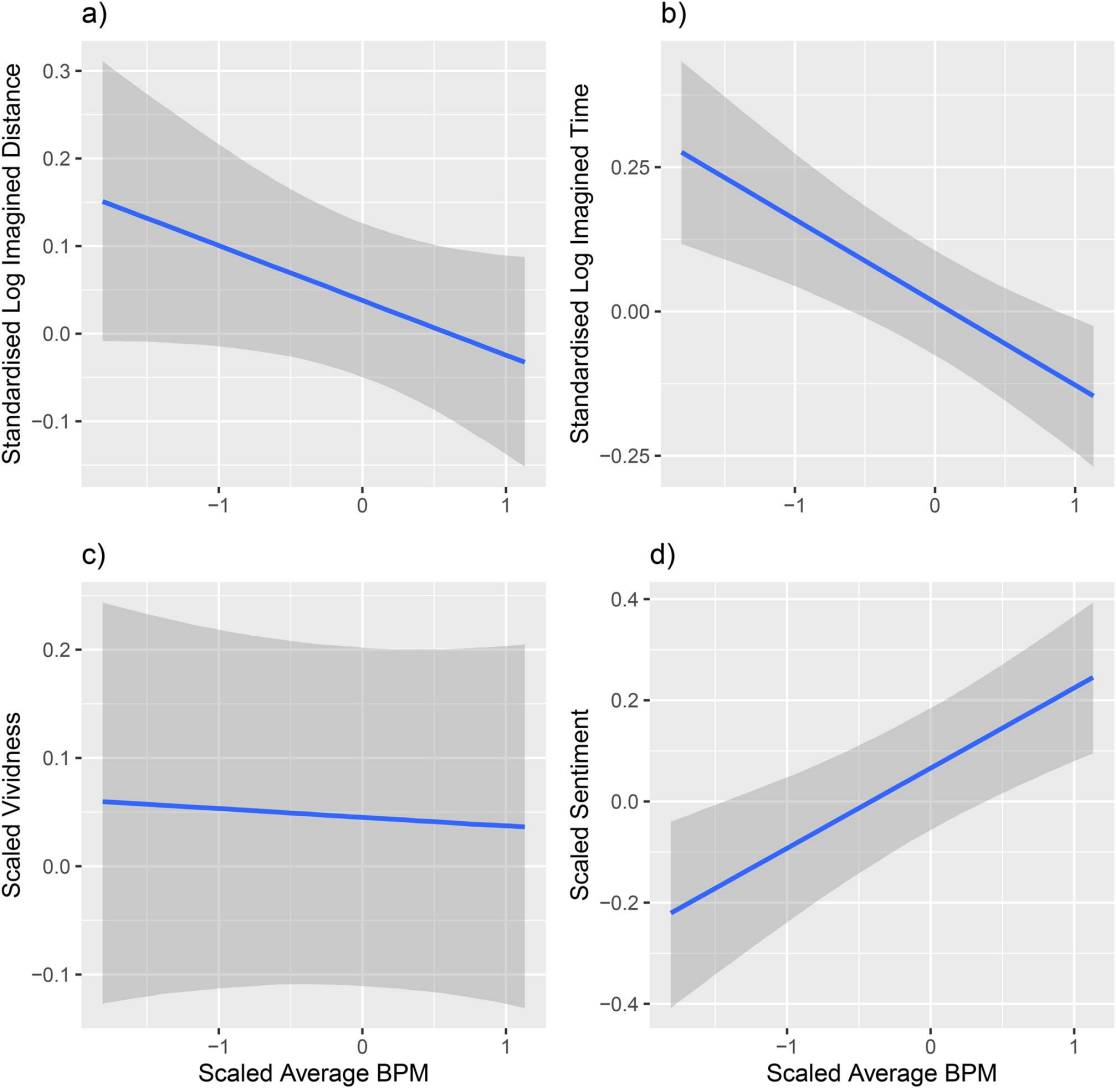
Standardised Log Imagined Distance (**a**) and time (**b**), as well as normalised vividness (**c**) and sentiment (**d**) as a function of scaled Average BPM. Higher BPM predicts lower imagined distance and time, as well as more positive sentiment of the imagined content. Vividness, however, is not predicted by BPM of the auditory inducers. Error bars show 95% CIs, note that a lot of the variability visible in this plot is accounted for in the models by the random effect structure.

Bayesian mixed effects models with the same random structure as above show strong evidence that higher Average BPM of the auditory inducers predicts lower Standardised Log Imagined Distance (*β* = − 0.06, *EE*_*β*_ = 0.04, *Odds* (*β* < 0) = 21.40*)*,* lower Standardised Log Imagined Time (*β* = − 0.14, *EE*_*β*_ = 0.04, *Odds* (*β* < 0) > 9999 *), more positive Sentiment (*β* = 0.16, *EE*_*β*_ = 0.04, *Odds* (*β* > 0) > 9999*), but does not have an effect on Vividness (*β* = − 0.01, *EE*_*β*_ = 0.03, *Odds* (*β* < 0) = 1.57) of the imagined content. This can be seen in Fig. 5, by the downward slopes in the top two panels, the upward slope in the bottom-right panel, and the flat prediction line in the bottom-left panel.

### Musical expertise is not directly reflected in the measures of imagined content

No predictive relationship between Musical Expertise or the Musical Expertise*Average BPM interaction and any of the measures of imagination utilised here was observed (all *Odds* < 3.78).

## Discussion

In the present study, we investigated whether music affects the vividness, sentiment, and content of directed imagination. Similar to prior studies on spontaneously evoked mind-wandering, all three dimensions were affected by the presence of background music.

Specifically, all musical auditory stimuli induced higher vividness ratings compared to the silent control condition. This effect was observed with all musical pieces and led on average to 2.7 times higher vividness of the imagined content compared to the silence condition. This finding supports the present use of music to facilitate recreational escapism^3^ or other directed instances of imagination, such as role play^32^. In a clinical setting, the present findings suggest that music may be used to systematically alter the vividness of imagination during exposure therapies, as vividness of the imagined content is a predictor of positive therapy outcome^34^. To put the present results into a concrete perspective, the Clinician-Administered PTSD Scale (CAPS)^53^ is the gold standard clinician-based assessment of Post-Traumatic Stress Disorder^54^. Mota et al.^34^ showed a decrease of CAPS scores after exposure therapy by 45.64 points averaged across 12 sessions. Using a 0–100 point scale of imagery vividness—which was also used in the present study—they show that as vividness of imagination during expose therapy increases, post-session CAPS scores are further reduced and this effect became even stronger as the number of therapy sessions increased. Based on the detailed reporting of results in Mota et al.^34^, the effect that music might have on exposure therapy outcome can be estimated. In the present study, on average, the musical auditory inducers led to 7.35 points higher vividness ratings on the same 0–100 point scale, with peak difference of up to 80 points. Based on Mota et al.^34^, this would mean in every session reducing post-treatment CAPS scores an additional 1 point on average, with up to an additional reduction of 9.86 points in individual cases. This would result in an estimated increase in efficacy by 2.2% on average in each exposure therapy session, and more than 20% in specific cases (see Supplement S4 for how exactly these numbers were obtained). According to Mota et al.'s^34^ findings, this effect would likely be weaker in the first sessions and even stronger in later sessions. Obviously, to what extent these numbers and effects generalise to a clinical treatment setting is an empirical question that awaits further study. However, it is important to note that the music used in the present study was also not optimised for use in a clinical setting. Prior research highlights the importance of using individualised music in therapy settings, which means that in a clinical setting, the therapeutic yield of using music as a supportive tool for exposure therapy may even be larger^55–57^. In summary, based on the current state of the literature and the present results, the potential of music to support exposure therapy is noteworthy.

Prior studies on non-directed mind-wandering and visual imagery have shown that music influences the sentiment of the imagined content^13,25^. Extending this finding to a directed imagination context suggests that music may be used as a catalyst in imagery rescripting therapies. Imagining a traumatic experience followed by creative imagined intervention that changes the outcome to a positive one has been shown to be an effective treatment in a plethora of conditions such as PTSD, Phobias, OCD, Nightmares, and Mood- as well as Personality Disorders^58^. The success of the therapy is contingent on the patient’s ability to introduce an imagined positive sentiment into a traumatic scenario, which often requires overcoming negative self-images and fighting intrusive images^59^. If music could be used to systematically influence the sentiment of the imagined content, then this could be used to facilitate the effect of the imagined intervention during imagery rescripting. Here, we demonstrate that music does indeed have an effect on the sentiment of imagined content. Within the current selection of stimuli, all music conditions induced more positive sentiments compared to the silence condition. However, this is likely subject to the precise stimuli utilised and other stimuli may have induced more negative sentiments. Indeed, the precise sentiment of the imagined content differed between auditory inducers, and future studies could investigate the role of enjoyment and musical preference in inducing imagined sentiment. Taken together, this suggests that well-selected music played during the intervention part of an imagery rescripting session may have therapeutic potential. Where the present study focused on a semantic analysis of the descriptions, the therapeutic potential could be further explored in the future with an in-depth thematic analysis of the descriptions provided by the participants^60^. Such an endeavour is supported by some recent research showing that within a given cultural context, there is some agreement about the prosaic narratives associated with musical excerpts^61^. Future research could explore whether this extends to music-induced imagination, by exploring whether thematic analysis identifies similarities in the themes within different participants’ imagined journeys when listening to the same musical pieces. However, the same study^61^ shows that between, rather than within, cultural contexts, agreement about prosaic narratives associated with musical excerpts is much less consistent. All stimuli in the present study were drawn from the Western music tradition, and the vast majority of participants lived in Western countries. Consequently, the present results would greatly benefit from replication using non-Western stimuli as well as a cross-cultural design. Replicating the present paradigm using a wide variety of different musical stimuli could generate the data to explore culture-dependent as well as culture-independent effects of music on imagination, whilst also allowing for further investigation into which structural and musical features mediate the induced imagined content. The present study makes a contribution in this direction by showing that the content is indeed subject to the choice of music, and that acoustic or psychoacoustic features, beyond musical structure, must be involved. This is demonstrated by the large systematic differences in induced sentiment between two music renditions of the same piece, which are structurally similar but differ across other features such as tempo (e.g., Bach-Furtwä﻿ngler vs. Bach-Gardiner). Future studies could replicate the present study using different musical materials to build up a large corpus of music and the imagined content it induces. Systematic feature extraction and analysis of such a corpus could provide great insights into causal relationships between musical features and induced imagination.

Such an endeavour may also be informed by the present findings about precise physical properties of the imagined content induced by the music. Specifically, prior literature demonstrated that orientation in space and time is retained in imagination^37,38^, a fact which was leveraged in the present paradigm by measuring imagined time passed and distance travelled within an imagined journey. The findings reveal systematic differences between auditory inducers. All but one auditory inducer induced larger distances travelled compared to the silence condition, whereas only half of the inducers showed greater passing of time during imagination. The results suggest that—comparable to their real analogues^62,63^—music can influence imagined distances and imagined time. The induced effect is systematic, but also highly specific to the inducer used. For example, the rendition of My Favorite Things by Mehldau, induced substantially more imagined time and distance compared to the rendition by Coltrane, whereas the two Bach renditions, which showed systematic difference in the sentiment measure, induced similar degrees of imagined distance and time. This suggests that given a large corpus—with the publicly available data from this study being one step into that direction—future studies may be able to extract the precise features responsible for inducing specific physical properties in the imagined content.

One such feature, tempo, was considered in the present study as a mediator. Similar to research on mind-wandering that uses less directed paradigms^18^, the present investigation reveals that tempo of the auditory inducers has a systematic effect on imagined content in directed imagination. Specifically, faster tempo predicted less imagined time passed and distance travelled, as well as more positive sentiment. However, tempo did not affect imagined vividness. It is important to note, that this study was not specifically designed to investigate the effect of tempo; instead, tempo was considered as a mediating variable. A future study could systematically change the tempo of a specific piece to gain further insight into the precise link between tempo and imagination in a more controlled fashion. Nevertheless, the present results allow the conclusion that some musical feature can selectively affect properties of the imagined content, which may allow for some degree of external control over imagination. Within a clinical setting, the present results highlight the supportive potential of music. For example, music could be used to increase the imagined amount of time passed whilst simulating a confrontation with a given stimulus during exposure therapy. Alternatively, music may be used as a supportive narrative device, for example, by putting larger amounts of distance between an imagined traumatic event and an imagined escape, or by inducing a more positive sentiment, whilst maintaining a similar degree of imagined vividness during an imagined intervention within a session of imagery rescripting therapy. Furthermore, based on the findings in the present study, such a clinical context would not need to be overly concerned with potential mediating factors of patients’ musical expertise, as they appear to be minimal.

Whilst the present results paint a clear picture about music’s ability to influence directed imagination, the driving forces behind the observed effects remain unclear. Music’s ability to generate predictions^64,65^, convey emotion^66–68^, as well as its resilience to forgetting^69,70^ paired with its capacity to evoke autobiographical memories^71^ would all be prime candidates. Additionally, cross-modal studies have shown that the neural activity in the early visual cortex can be used to classify heard and imagined sounds, as well as their emotional connotation^72,73^. Whereas the evolutionary purpose of such a neural connection may be to provide a categorical prediction for anticipated incoming visual input^72^, the existence of this information channel may be involved in the effects observed in the present study. The precise mechanisms or combination of mechanisms underpinning the effects of music on imagination observed here awaits future scientific attention.

The present study focused on the effect of music on directed imagination, however, prior literature suggests that some of the auditory features that trigger emotional responses to music also evoke emotional responses to non-musical sounds^74^. Future research could systematically investigate auditory features involved in mediating imagination and compare effects observed in stimuli such as noise or natural sounds with those observed in music. Extending the present results to non-musical stimuli may help in generalising beneficial stress-reducing effects observed in natural sounds within virtual environments^75^ to imagined environments. Furthermore, a deeper understanding of the precise features, both musical and non-musical, involved in mediating imagination, may pave the way for automatic sound generation systems, that produce an acoustic environment based on user specified effects on imagined vividness and content.

## Conclusion

The present study offers a novel paradigm to investigate directed imagination and highlights the recreational benefits of listening to music in terms of escapism^3^, as well as music’s potential to support Cognitive Behavioural Therapy, specifically Exposure Therapy and Imagery Rescripting, which deploy directed imagination as a clinical tool for the detection and treatment of mental health conditions^8,76–79^.

## Method

### Participants

Data of 100 participants (34 females, 65 males, 1 preferred not to disclose; *M*_*Age*_ = 28.27, *SD*_*Age*_ = 9.85, *Range* = 18 to 55 years) were collected through the online platform Prolific academia. The experiment was approved by the Ethics Committee of the École Polytechnique Fédérale de Lausanne (Approval 009-2019/21.02.2019), all participants provided informed consent, and the experiment was conducted in accordance with the declaration of Helsinki. Participants were required to be fluent in English, and be able to play back the audio. No participants reported any hearing impairments. Three participants did not provide any responses during the task. As a result, their data was discarded, and three new participants were collected. Participants were located in Europe (88%), North America (7%), Africa (2%), South America (2%), and Asia (1%) with a wide spread of musical expertise (*M* = 12.05, *SD* = 9.73, *Range* = 0 to 39, on the 0-to-42 point musical training subscale of the Gold-MSI which is assessed through 7 questions). Participation was reimbursed with CHF 12.

### Imagination task

In the imagination task, participants were presented with a visual inducer, that functioned as a common reference framework for participants’ responses by providing a common starting point and direction for participants’ imagined journeys. The visual inducer constituted a video of a figure ascending a small hill (Fig. 1a). Once the figure reached the top of the hill, a vague landmark appeared in the far distance (i.e., illuminated mountain in Fig. 1b). The visual inducer originated from the video game “Journey” with written permission of Jenova Chen, CEO of *ThatGameCompany* (https://thatgamecompany.com).

After a period of 15 s from the beginning of the video, participants heard a gong-sound and were prompted to close their eyes and imagine a continuation of the figure’s journey towards the landmark. After this point, no video was played, only a black screen with white lettering stating “Please close your eyes”. One minute and thirty seconds later, the gong-sound was played again, signalling participants to open their eyes again and answer a series of questions. The possible inputs varied depending on the question and are indicated in brackets below:

1. How much time really passed between the two gong sounds? (Minutes, seconds)
2. How far away do you estimate the mountain to be at the beginning of the journey? (Kilometres, meters)
3. How much time passed in your imagination? (Years, months, days, hours, minutes, seconds)
4. How far did you travel in your imagination? (Kilometres, meters)
5. How vivid (clear) was the imagery you experienced compared to experiences in real life? (Numeric response between 0 (not very clear) and 100 (very clear))
6. Please describe your imagination in as much detail as possible. (Free format text box)

Participants were informed to include any time or distance skips into their estimates. For example, if they imagined a journey of an hour, then skipped ahead a year, and imagined a continuation of the journey a year later, they were instructed to include the year into their estimate. The large resolution in question 3 was necessary, as pilot testing showed that some participants imagined differences between journeys on very small time-scales (seconds), whereas other participants reported differences between journeys on very large time-scales (years). Questions 1 and 2 were not analysed and only functioned to highlight the difference between real and imagined time and space, and indeed no participant consistently provided the same response to the real and imagined question about time or space. All instructions appeared on the screen and participants provided typed responses. Each participant performed the same task in seven trials. Each trial was identical, with the exception of the auditory inducer (see “Auditory inducer” section). Participants were asked to “please treat every repetition independently from one another. You can imagine a similar or a totally different journey every time. This is entirely up to you’ and informed that ‘there are no restrictions on your imagination, but please always keep the mountain in sight. This is because after your imagination you will be asked a few questions about the time and distance travelled in your imagination, and the mountain can help you orientate.” The precise wording and sequencing of all instructions can be found in the Supplement S2. After the last imagination trial, participants were asked to fill out the Goldsmith Musical Sophistication Index, however, only the musical training subscales was utilised in the present study^80^. The data was used to test for potential mediating factors of musical expertise on the imagination measures used in the present task. The entire experiment took between 30 and 60 min to complete, largely depending on the depth of detail provided by the participants in the open-ended description of the imagined journey (Question 6).

Before collecting data in the main experiment, the task was piloted with a convenience sample of 33 participants, recruited through student mailing lists. After showing promising results, the study was released for the larger data collection. The two datasets were not combined, as the pilot study did not include the vividness question nor the musical sophistication questionnaire.

### Auditory inducer

During this imagination task, auditory inducers consisting of six musical stimuli as well as a control condition in form of silence were used. In each trial, participants listened to one the auditory inducers. The order of the auditory inducers was fully randomised between participants, but each participant was presented with each inducer. The six musical stimuli consisted of three musical pieces (J. S. Bach, Chorale ‘O Haupt voll Blut und Wunden’, from Matthäus Passion BWV244; C. Debussy, ‘Tarantelle Styrienne’ L. 69; R. Rodgers, ‘My Favorite Things’), each presented in two renditions. The stimuli were specifically selected to provide easy access to multiple renditions, which provides valuable insights into the commonalities and differences introduced by the differences of the respective renditions. Specifically, the two renditions of ‘O Haupt voll Blut und Wunden’ (by J. E. Gardiner, Monteverdi Choir, English Baroque Soloists, 1989 and W. Furtwängler, Wiener Singakademie, Wiener Philarmoniker, 1954) are identical in terms of musical structure, notes, and instrumentation, but vary in terms of performance-related features such as tempo, agogic, articulation, vocal and instrumental technique, and adoption of historically informed performance practices. The two renditions of ‘Tarantelle Styrienne’ (Renditions for piano, J.-Y. Thibaudet, 2000, and in the orchestration by Ravel, L. Slatkin, Orchestre National de Lyon, 2016) are different instrumentations of an original piano score by Claude Debussy: they vary in instrumentation (piano vs. symphonic orchestra), but the musical structure is identical. The two renditions of ‘My Favorite Things’ (rendition by The Jon Coltrane Quartet, 1961, a jazz quartet with a solo soprano saxophone, and a piano rendition by B. Mehldau, live concert in Vienna, 2010) are jazz performances with a strong improvisational component: as a consequence, while they share the same core thematic materials, they differ both in instrumentation as well as in local harmonic, rhythmic and melodic features as well as large-scale musical structure. Obviously, the precise pattern of results will always be subject to the inducers chosen. Consequently, the present study should be taken as a proof-of-concept encouraging replication with other auditory stimuli to specifically target features of interest. All stimuli were loudness normalised to the common value of − 23 ± 5*10^–7^ LUFS, as per EBU R-128^81^ with the pyloudnorm Python library^82^, and further detail as well as their sources are available in Supplement S1.

## Supplementary Information

**Supplementary Information**.
