# Supplementary material for: Music influences vividness and content of imagined journeys in a directed visual imagery task
Source: Sci Rep. 2021 Aug 6;11:15990. doi: 10.1038/s41598-021-95260-8 (PMC8346606; doi:10.1038/s41598-021-95260-8)
Supplement: Supplementary file 1 — Supplementary Information. [file 41598_2021_95260_MOESM1_ESM.zip › Online Supplement/S4 Estimates Based on Mota et al/S4 Estimates based on Mota et al (2015).pdf]

S4. Estimates based on Mota et al (2015)

The overall treatment effect of the exposure therapy was obtained from Table 2 in Mota et al. (2015). The vividness effect was taken from the most parsimonious model shown in Table 4 of the same article. As Mota et al use a square-root transformation (i.g., sqrt(max(x)-x)) to adjust for the negative screw in their vividness distribution, the same transformation was applied to the vividness ratings in the present data before comparison. For example, for the man difference of 7.35 in vividness between 60.28 (musical auditory inducers) and 52.93 (silence condition) would translate to a sqrt(101-60.28)- sqrt(101-52.93) = -.552 difference on the transformed vividness scale. Multiplying this with the effect size obtained from Mota et al (i.g., a change of 1.82 CAPS points per one point increase on the transformed vividness scale; note that the signs in Table 4 are reverted for ease of read) means an average reduction of -.552*1.82 = -1.005 CAPS points. The peak difference was calculated in the same way using the maximal difference an individual participant showed between the silence and a musical auditory inducer condition. All Supplemental Online Material is available on https://osf.io/xemhk/ .
