# Supplementary material for: Music influences vividness and content of imagined journeys in a directed visual imagery task
Source: Sci Rep. 2021 Aug 6;11:15990. doi: 10.1038/s41598-021-95260-8 (PMC8346606; doi:10.1038/s41598-021-95260-8)
Supplement: Supplementary file 1 — Supplementary Information. [file 41598_2021_95260_MOESM1_ESM.zip › Online Supplement/S1 Stimulus Overview/S1-Stimulus-Overview.pdf]

Supplemental Material

S1. Stimuli Overview

Table S1. Overview of the stimuli used in the present study

Index	Title	Composer	Performer	Commercial Example Link	Streaming Example Link	Avg. BPM
Bach-Gardiner	"O Haupt voll Blut und Wunden", from Matthäus Passion BWV244	J. S. Bach	J. E. Gardiner (Monteverdi Choir, English Baroque Soloists, 1989)	https://www.deccaclassics.com/de/katalog/produkte/bach-matthaeus-passion-gardiner-7874	https://open.spotify.com/track/21fzjpUxYcznbyATUAxPTh?si=stnoijOEQOCidMWiGesmTQ	78
Bach-Furtwangler	"O Haupt voll Blut und Wunden", from Matthäus Passion BWV244	J. S. Bach	W. Furtwängler (Wiener Singakademie, Wiener Philarmoniker, 1954)	https://uatprod.arkivmusic.com/products/wilhelm-furtwangler-wiener-konzerte-1944-1954-18-257489	https://open.spotify.com/track/6reLmioSkOjiCEFKQBfYpa?si=97nLFhMHSmWoey_vXOl81Q	32
Debussy-Thibaudet	"Tarantelle Styrienne" L. 69	C. Debussy	J.-Y. Thibaudet (2000)	https://www.deccaclassics.com/en/catalogue/products/debussy-piano-works-vol-2-thibaudet-3651	https://open.spotify.com/track/0p28R47fiy3LIdofOkeTge?si=A6NSSxf2RnKVf-vjkpuQsQ	146
Debussy-Ravel	"Tarantelle Styrienne" L. 69	C. Debussy	L. Slatkin (Orchestre National de Lyon, 2016)	https://www.naxos.com/catalogue/item.asp?item_code=8.573124	https://open.spotify.com/track/4kjfuIt2uz3CMmZ2HUsw0c?si=0M3erIqHTbOIQm1l0x6RtQ	135
MFT-Coltrane	"My favourite things"	R. Rodgers	The Jon Coltrane Quartet (1961)	https://www.amazon.com/My-Favorite-Things-John-Coltrane/dp/B00000348A	https://www.youtube.com/watch?v=qWG2dsXV5HI	165
MFT-Mehldau	"My favourite things"	R. Rodgers	B. Mehldau (live 2010)	https://catalogue.bnf.fr/ark:/12148/cb42310577f	https://www.youtube.com/watch?v=XDDr5TwpJYY	127

Note. Only the first 105 second of each stimulus was played. The average BPM refers to these sections of the pieces.

Due to copyright reason, the original audio files cannot be shared here.

S2. Instructions

Online Supplement S2 contains two files. The file ‘S2 Instructions.xlsx’ contains the order and precise wording of the task instructions. The file ‘S2 Questions.xlsx’ contains the order and precise wording of the questions posed to the participants.
