# Supplementary material for: Music influences vividness and content of imagined journeys in a directed visual imagery task
Source: Sci Rep. 2021 Aug 6;11:15990. doi: 10.1038/s41598-021-95260-8 (PMC8346606; doi:10.1038/s41598-021-95260-8)
Supplement: Supplementary file 1 — Supplementary Information. [file 41598_2021_95260_MOESM1_ESM.zip › Online Supplement/S3 Data and Scripts/S3 Analysis/pVividnessSig.pdf]

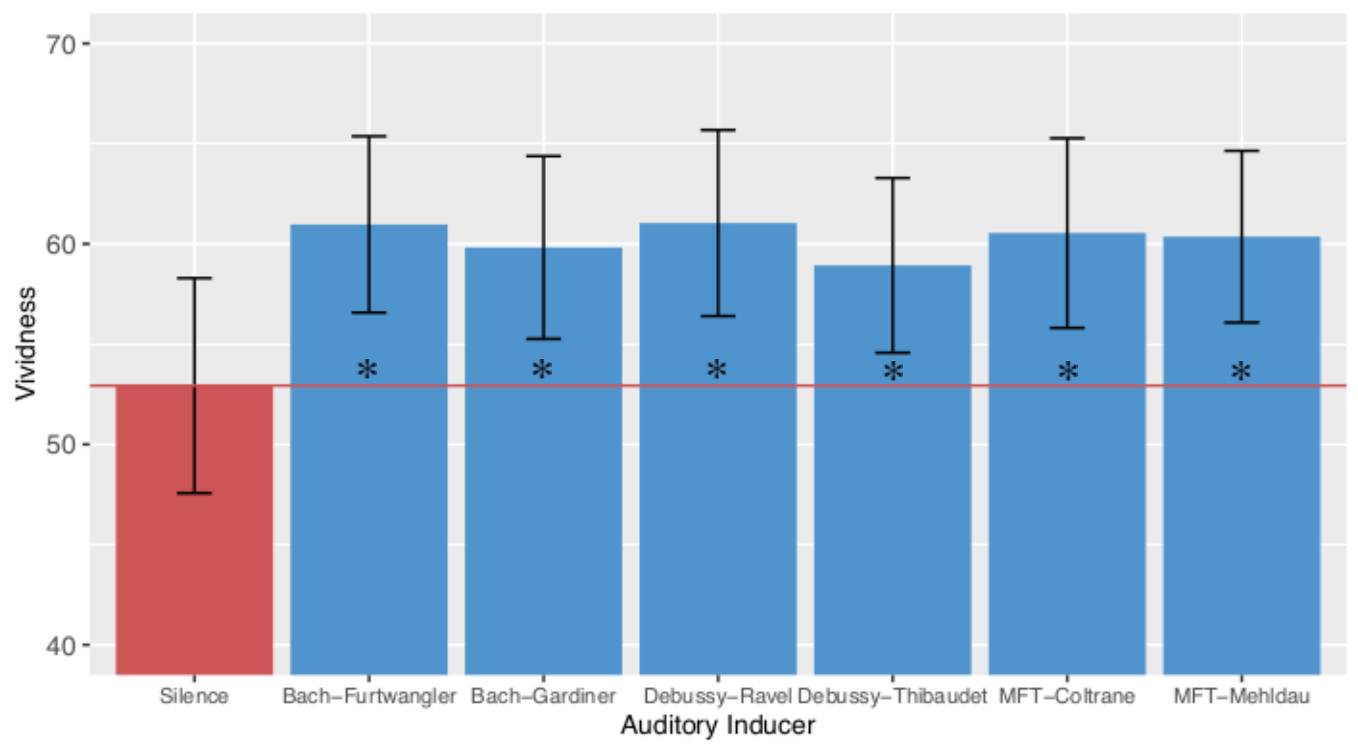
70
60
50
40
Vividness
*
*
*
*
*
*
Silence
Bach–Furtwangler
Bach–Gardiner
Debussy–Ravel
Debussy–Thibaudet
MFT–Coltrane
MFT–Mehldau
Auditory Inducer
