# Supplementary material for: Music influences vividness and content of imagined journeys in a directed visual imagery task
Source: Sci Rep. 2021 Aug 6;11:15990. doi: 10.1038/s41598-021-95260-8 (PMC8346606; doi:10.1038/s41598-021-95260-8)
Supplement: Supplementary file 1 — Supplementary Information. [file 41598_2021_95260_MOESM1_ESM.zip › Online Supplement/S3 Data and Scripts/S3 Analysis/FeatureTempoPlot.pdf]

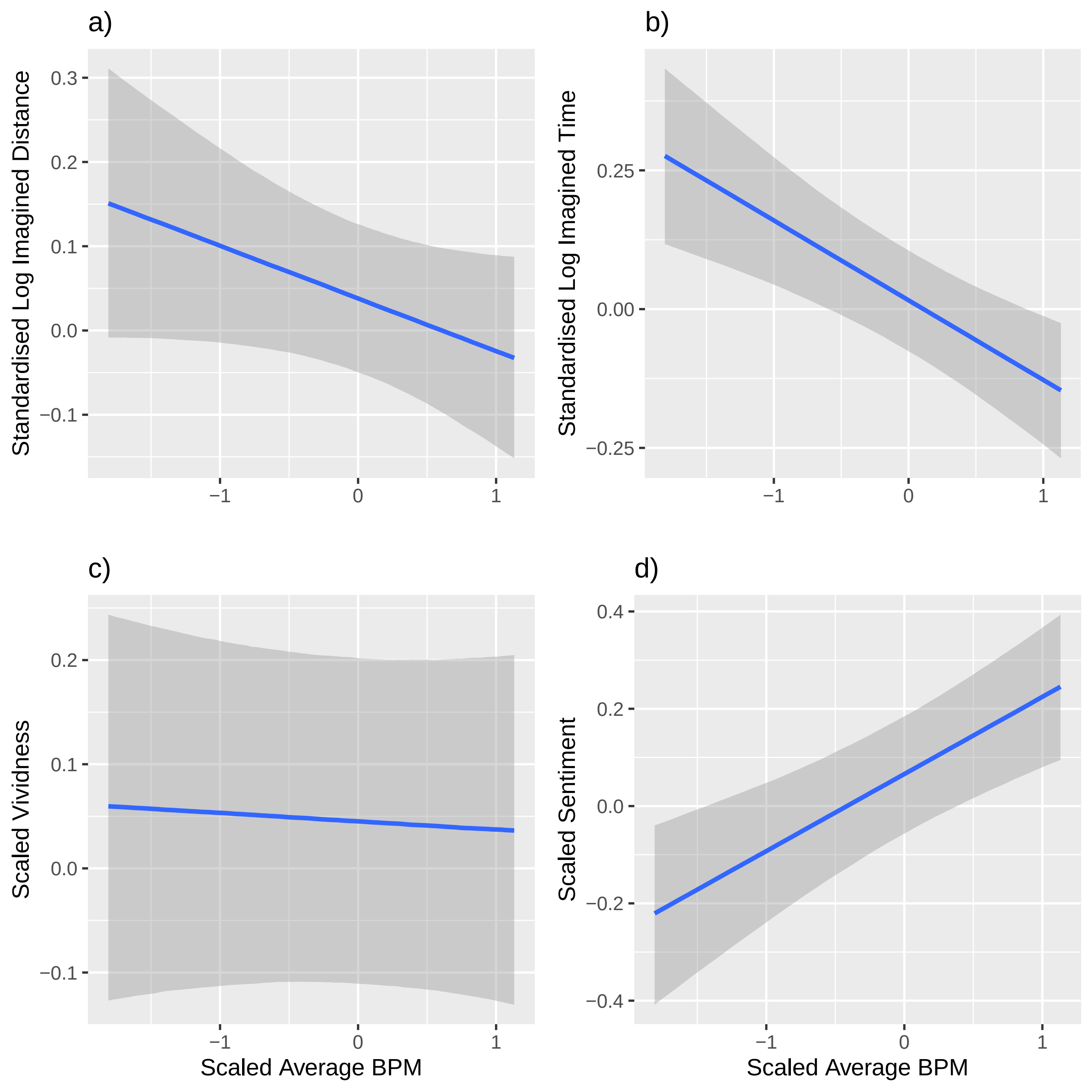

a)
Standardised Log Imagined Distance
0.3
0.2
0.1
0.0
−0.1
−1
0
1
b)
Standardised Log Imagined Time
0.25
0.00
−0.25
−1
0
1
c)
Scaled Vividness
0.2
0.1
0.0
−0.1
−1
0
1
Scaled Average BPM
d)
Scaled Sentiment
0.4
0.2
0.0
−0.2
−0.4
−1
0
1
Scaled Average BPM
