# Supplementary material for: Music influences vividness and content of imagined journeys in a directed visual imagery task
Source: Sci Rep. 2021 Aug 6;11:15990. doi: 10.1038/s41598-021-95260-8 (PMC8346606; doi:10.1038/s41598-021-95260-8)
Supplement: Supplementary file 1 — Supplementary Information. [file 41598_2021_95260_MOESM1_ESM.zip › Online Supplement/S3 Data and Scripts/S3 Analysis/pSentiment.pdf]

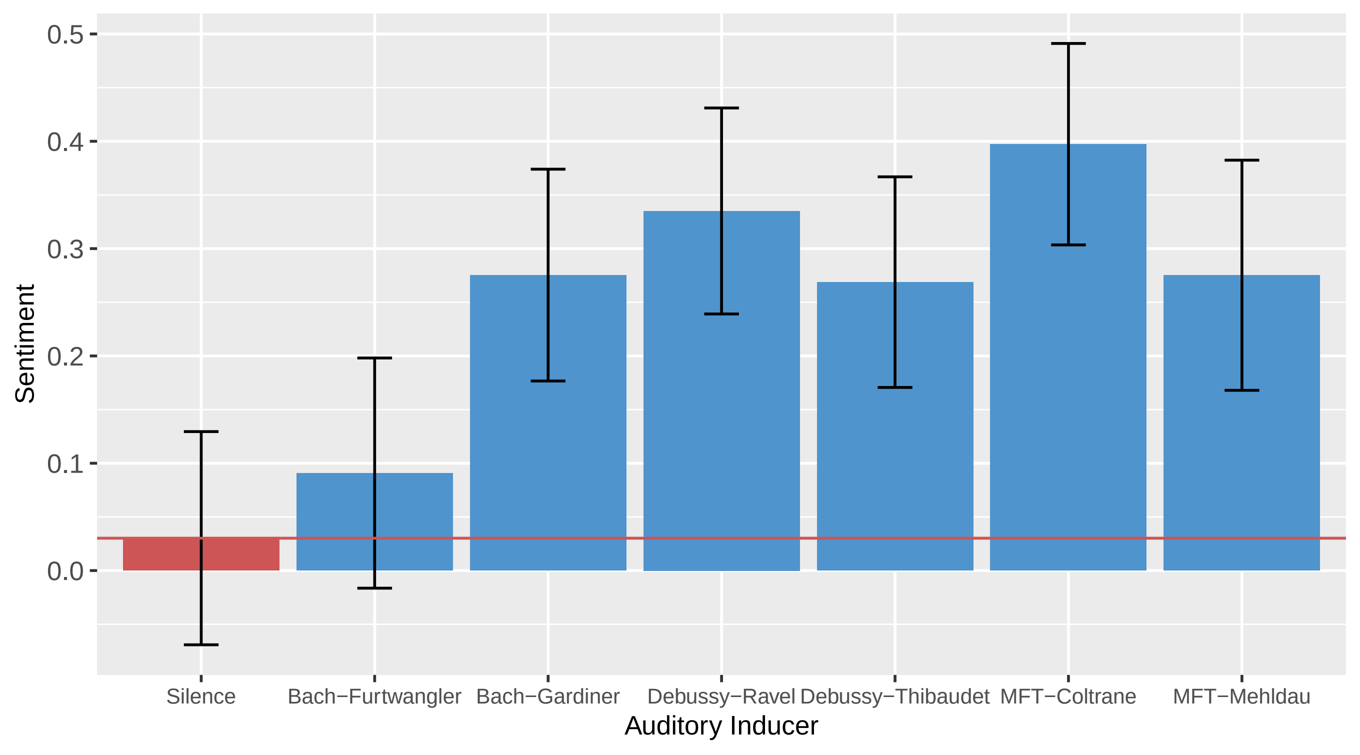

0.5
0.4
0.3
0.2
0.1
0.0
Sentiment
Silence
Bach−Furtwangler
Bach−Gardiner
Debussy−Ravel
Debussy−Thibaudet
MFT−Coltrane
MFT−Mehldau
Auditory Inducer
